# Role of S100 family proteins in colorectal cancer (CRC): an overview of their potential function as new biomarkers and therapeutic agents

**DOI:** 10.1017/erm.2025.10019

**Published:** 2025-09-23

**Authors:** Hamideh Raeisi, Leili Rejali, Nayeralsadat Fatemi, Amir Sadeghi, Zahra Sadeghloo, Mohammad Reza Zali, Ehsan Nazemalhosseini Mojarad

**Affiliations:** 1 Gastroenterology and Liver Diseases Research Centre, Research Institute for Gastroenterology and Liver Diseases, Shahid Beheshti University of Medical Sciences, Tehran, Iran; 2 Basic and Molecular Epidemiology of Gastrointestinal Disorders Research Centre, Research Institute for Gastroenterology and Liver Diseases, Shahid Beheshti University of Medical Sciences, Tehran, Iran

**Keywords:** biomarkers, CRC, drug resistance, S100 proteins, therapeutics, tumourigenesis

## Abstract

Colorectal cancer (CRC) is the second deadliest cancer worldwide, posing a great threat to human health and a social burden. Various genetic and epigenetic alterations can activate tumourigenesis-related signalling pathways, leading to CRC development and progression. Over the past two decades, the understanding of the role of S100 family proteins in different types of cancer has received great attention. S100 proteins, as intracellular and extracellular, play important roles in regulating various cellular processes, such as calcium homeostasis, apoptosis, tumour cell proliferation, invasion and motility. It is well documented that alteration in expression of S100 proteins can be associated with tumourigenesis and cancer progression. These proteins play important roles in CRC carcinogenesis by activating different signalling pathways, especially the nuclear factor kappa B (NF-κB) signalling pathway, which is involved in cell proliferation, invasion and migration. In this review, we describe the functions of S100 proteins in the context of inflammation, tumourigenesis, cancer progression, metastasis, and drug resistance in CRC. We also discuss the potential of targeting different S100 proteins as prognostic factors and therapeutic agents for CRC treatment. This narrative review will increase our understanding of the role of S100 proteins in the progression of CRC and provide insights into the use of S100 proteins as new biomarkers and therapeutic targets for CRC therapy.

## Introduction

Colorectal cancer (CRC) is the second most fatal cancer and the third most prevalent malignant tumour worldwide, causing a highly heterogeneous malignancy in colon and rectum tissues (Ref. 1). Surveillance data from 2018 estimated the number of CRCs at approximately 1.8 million new cases and 881,000 deaths, accounting for nearly 10% of new cancer cases and cancer deaths worldwide (Ref. 2). CRC is multifactorial in origin; for example, age (>50 years) and genetics (family history of polyps and CRC) are the main risk factors for this cancer (Ref. 3). Several environmental factors, such as obesity, smoking, alcohol consumption, high-fat/low-fibre diets, and chronic intestinal inflammation, may increase the risk of CRC (Refs. 4, 5). CRC typically develops in several stages, from polyps and adenocarcinoma to malignant tumours (Ref. 6), and progresses in four steps, including initiation, promotion, progression and metastasis (Ref. 7). Initiation of CRC involves irreversible genetic alteration, resulting in neoplastic transformation. In promotion, cell proliferation leads to abnormal growth. The progression phase contains aberrations of genetic/epigenetic that convert benign cells to malignant cells, resulting in aggressive characteristics and metastasis. Eventually, the potential spread to other organs of the body through the blood and lymph nodes occurs during the metastasis phase (Refs. 1, 6).

So far, different classifications have identified CRC subtypes with distinctive genotypic and phenotypic traits. In this regard, the CRC Subtyping Consortium (CRCSC) established a classification system for CRCs in 2015 and grouped them into four consensus molecular subtypes (CMS) based on distinct gene expression patterns (Ref. 8). In this classification, CMS1 (MSI-immune, 14%) is characterised by high microsatellite instability (MSI), low chromosomal instability (CIN), hypermutation, hypermethylation and strong immune infiltration and activation. CMS2 (canonical, 37%) is featured by high CIN, high epithelial differentiation, and a large number of somatic copy number alterations (SCNAs). CMS3 (metabolic, 13%) is distinguished by the dysregulation of genes associated with cellular metabolic processes, the presence of kirsten rat sarcoma viral oncogene homologue (KRAS) mutations, and CpG island methylator phenotype-low (CIMP-low). CMS4 (mesenchymal, 23%) is characterised by the widespread activation of transforming growth factor-β (TGF-β) signalling, and the overexpression of genes involved in inflammation, stromal invasion and angiogenesis. Notably, patients with CMS4 have worse overall and relapse-free survival rates than those with the other three subtypes. In addition to CMSs, an unclassified group (mixed features, 13%) is characterised by transition phenotype or intratumoural heterogeneity (Refs. 8, 9).

Using transcriptional analysis, a classification based on tumour biology regardless of mutational status was published in 2023 and characterised three pathway-derived subtypes (PDSs) for CRC, including PDS1 tumours with canonical/LGR5+ stem-rich cells, upregulated cell cycle pathways, and good prognosis; PDS2 tumours with regenerative/ANXA1+ stem-rich cells, upregulated inflammatory and immune signalling pathways and elevated stromal and immune tumour microenvironmental lineages; and PDS3 tumours with decreased stem cell populations and increased differentiated lineages and displayed the worst prognosis in locally advanced disease. In line with CMS associations, CMS groups can be combined in PDS subtypes; accordingly, the inflammatory/stromal CMS1 and CMS4 tumours are distinguished as PDS2, and CMS2 and CMS3 tumours are characterised as PDS1 and PDS3 (Ref. 10). Remarkably, CMSs and PDSs are robust classifications for CRC, providing deeper insights into CRC biology.

In the past two decades, various epidemiological and experimental studies have suggested that changes in the expression of some proteins, such as S100 proteins, may increase the probability of occurrence of various cancers (Refs.^11^–^15^). S100 proteins belong to the largest subfamily of EF-hand-type calcium-binding proteins (Ref. 16). S100 proteins can interact with various protein targets, resulting in the activation of different intracellular and extracellular cascades (Ref. 17). Thus, these proteins influence several functions in cellular processes, such as Ca^2+^ homeostasis, inflammation, apoptosis, tumourigenesis, metastasis and drug resistance (Ref. 16). Accumulated data have demonstrated that different members of the S100 family are involved in the activation of various signalling pathways related to cancer and affect the expression of genes related to cancer progression, such as nuclear factor-kappa B (NF-κB), β-catenin and p53 (Refs. 18–21).

This review discusses the roles of S100 proteins in inflammation, cancer development and progression and resistance to chemotherapy. In addition, the potential of different S100 proteins as prognostic biomarkers and therapeutic agents for CRC is summarised. This narrative review provides a comprehensive overview of the importance of S100 proteins in tumourigenesis, their potential as diagnostic agents for managing cancer and their role as therapeutic agents in suppressing cancer development and progression.

## Function of S100 proteins

S100 is a subfamily of low-molecular-weight calcium-binding proteins with more than 20 known human members with similar structures but different functions (Ref. 16). These proteins were named ‘S100’ because they exhibit 100% solubility in ammonium sulfate at neutral pH (Ref. 22). Among human S100 genes, group A is located in the chromosomal regions of 1q21, whereas other members are located in different chromosomal regions (Ref. 17), including S100B (21q22), S100G (Xp22), S100P (4p16) and S100Z (5q14) (Ref. 23). All S100 proteins have a similar molecular weight of about 10–12 KDa with 25–65% similarity in their amino acid sequence (Refs. 16, 17). The functions of S100 proteins are regulated via two different mechanisms: (1) binding of metal ions Ca^2+^, which activates the protein to bind partner proteins, and transition metals (e.g., Zn^2+^, Cu^2+^, Mn^2+^), which serve in antimicrobial functions; (2) formation of homo- and hetero-oligomers (Ref. 16). S100 proteins consist of two helix–loop–helix structural motifs (known as EF-hands (EF-1 and EF-2)) connected through a flexible hinge region in the center and flanked by hydrophobic residues at the N- and C-termini (Refs. 16, 17). S100 proteins (except S100G) are obligate dimers with a contiguous hydrophobic core (Ref. 22) and also form higher-order oligomers typically mediated by metal binding. The most highly characterised S100 protein heterodimer is S100A8/S100A9 (calprotectin), a crucial mediator of inflammatory and immune responses (Ref. 24). Other examples of S100 protein heterodimeric complexes include S100A1/ S100B, S100A11/ S100B, S100A1/ S100A4 and S100A1/S100P (Ref. 25).

**Figure 1.**
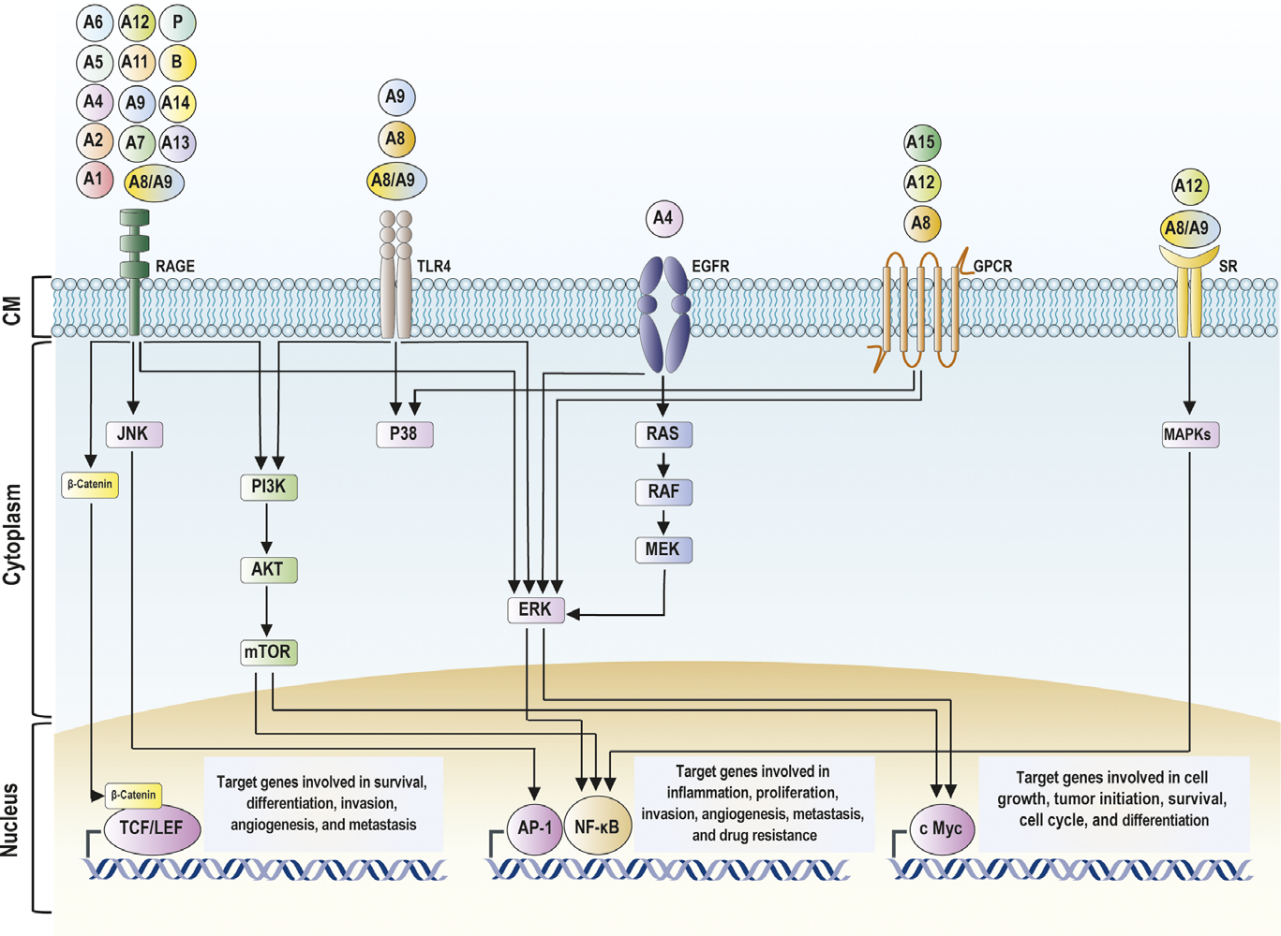
Schematic diagram of the signalling pathways activated by extracellular S100 proteins. These proteins bind to different cell surface receptors, including RAGE, TLR4, EGFR, GPCR, and SR, and subsequently activate several signalling pathways involved in cancer progression. RAGE-mediated signalling activates MAPK, PI3K/AKT, and β-catenin pathways. TLR4-mediated signalling activates MAPK and PI3K/AKT pathways. GPCR, EGFR-, and SR-mediated signalling activate MAPK pathways. The activation of these signalling pathways subsequently activates signalling cascades related to cancer progression, especially NF-κB signalling pathways, leading to the upregulation of genes involved in cell survival, proliferation, differentiation, invasion, metastasis, and drug resistance. Abbreviations: AKT, protein kinase B; AP-1, activator protein 1; c-myc, cellular myelocytomatosis oncogene; EGFR, epidermal growth factor receptor; ERK, extracellular signalling-related kinase; GPCR, G protein coupled receptor; JNK, c-Jun N-terminal kinase; MEK, mitogen-activated protein kinase kinase; mTOR, mammalian target of rapamycin; NF-κB, nuclear factor κB; PI3K, phosphatidyl inositol 3 phosphate; RAGE, receptor for advanced glycation end products; RAF, rapidly accelerated fibrosarcoma; RAS, rat sarcoma virus; SR, scavenger receptor; TCF/LEF, T-cell factor/lymphoid enhancer factor; TLR4, toll-like receptor 4.

S100 proteins are multifunctional with intracellular and extracellular activities in different cell types. Intracellular S100 proteins are associated with the regulation of various cellular processes, such as calcium homeostasis, cell growth, cell cycle, cytoskeletal components, phosphorylation and modulation of transcriptional factors (Ref. 16). In the extracellular milieu, S100 proteins can act in a cytokine-like manner, binding to cell surface receptors such as the receptor for advanced glycation end products (RAGE), toll-like receptors (TLRs), epidermal growth factor receptor (EGFR), G protein-coupled receptors (GPCR) and scavenger receptors (SRs), which activate different signalling pathways related to cell proliferation and differentiation, particularly the NF-κB signalling pathway (Figure 1) (Refs. 17, 21, 26, 27).

Different S100 proteins can bind to the same targets because of their high sequence and structural similarity. For example, S100A1, S100A6 and S100B interact with annexin 6 (ANXA6), and S100A1, S100A2, S100A4, and S100B can interact with p53, a tumour suppressor (Ref. 17). Despite these similarities in binding partners, the most variable portion of S100 proteins is the regions exposed upon Ca^2+^ binding, which does provide a degree of selectivity for target proteins (Ref. 17). Selectivity is provided by differences in the protein surface and the distribution of hydrophobic and charged residues (Ref. 16). This in turn provides the functional diversity, for example, the involvement of S100A1, S100A4, S100A6 and S100A9 in cytoskeleton assembly, involvement of S100A4, S100A11, S100A14 and S100B in DNA repair and transcriptional regulation, involvement of S100A6, S100A8/S100A9 and S100B in cell differentiation, involvement of S100A4, S100A8/S100A9, S100A12 and S100A13 in inflammation state, and involvement of S100A6, S100A9 and S100B in cell growth and migration have been reported in different studies (Refs. 16, 19, 28–34). Some functions of S100 proteins in cancer progression are described in the following.

## Roles of S100 proteins in cancer

The upregulation of S100 proteins in different diseases has attracted considerable attention, particularly in oncological research (Refs. 12, 17, 47, 52, 71, 79). Most of the S100 protein genes are located in the human chromosomal region of 1q21, and genomic instability of this region is often observed in various tumours (Refs. 80, 81). Multiple lines of evidence have demonstrated that altered expression of S100 proteins, typically upregulation, can be associated with tumour development, angiogenesis and metastasis (Refs. 28, 82, 83). In addition, several S100 proteins appear to contribute to drug resistance and may mediate responses to chemotherapy (Refs. 61, 84, 85). S100 proteins are expressed in a tissue-dependent manner, and the function of different S100 proteins may be different in various cancers (Ref. 17). For example, S100A2 acts as a tumour suppressor in oral cancer, while it facilitates tumour growth in lung cancer (Refs. 33, 86). Pathologic S100 signalling has been explored in melanoma, breast, lung and ovarian cancers (Refs. 87–90). Moreover, multifunctional roles of S100 proteins in gastrointestinal (GI) tract cancers have been identified (Table 1).

**Table 1.** S100 proteins: characterisation and function in GI tract cancers

S100 proteins	Other name	Interacting receptors	Major cells expressing	Setting	Functions	Expression	References
S100A1	S100α	RAGE	Macrophage, dendritic, epithelial, and endothelial cells	HCC tissue samples HCC model (*in vitro*)	Induction of cell proliferation Inhibition of cell apoptosis Ca^2+^ homeostasis	Upregulated in HCC	(Ref. 35)
S100A2	S100L, CaN19	RAGE	Cancer cells	PDAC tissue samples EC models (*in vitro*) CRC tissue samples HCC model (*in vitro*) GC tissue samples	Induction of cell proliferation, invasion, and migration Tumour suppression Chemotaxis	Upregulated in EC, PDAC, CRC, HCC Downregulated in GC A biomarker for progression/metastasis or prognosis in PDAC	(Refs. 12, 36–39)
S100A3	S100E	RARα	Endothelial, epithelial, and lymphatic cells	CRC tissue samples GC tissue samples HCC model (*in vitro*)	Induction of cell differentiation and invasion	Upregulated in CRC, GC, HCC	(Refs. 40–42)
S100A4	Metastasin1	RAGE, EGFR	Tumour cells, neutrophils, macrophages, and T cells	EC tissue samples EC model (*in vitro*) PDAC tissue samples GC tissue samples GC model (*in vitro*) CRC tissue samples HCC models (*in vitro* and *in vivo*)	Induction of cell proliferation, differentiation, motility, invasion, migration, angiogenesis, tumour Involvement in microenvironment formation	Upregulated in EC, PDAC, GC, CRC, HCC A biomarker for progression/metastasis or prognosis in multiple cancers	(Refs. 14, 43–46)
S100A6	Calcyclin	RAGE	Neutrophils, macrophages, and NK cells	PDAC tissue samples PDAC model (*in vitro*) HCC models (*in vitro*) GC serum samples CRC tissue samples CRC model (*in vitro*)	Induction of cell proliferation, differentiation, motility, invasion, migration Ca^2+^ homeostasis Antibacterial activity	Upregulated in PDAC, HCC, GC, CRC A diagnostic marker for PDAC and GC	(Refs. 15, 47–49)
S100A7	Psoriasin1	RAGE	Epithelial cells and neutrophils	EC serum and tissue samples PDAC models (*in vitro*)	Induction of cell proliferation, differentiation, motility, invasion, migration	Upregulated in EC and PDAC Potential biomarker for EC	(Refs. 50, 51)
S100A8/A9 complex	Calprotectin	RAGE, TLR4, GPCR, SR	Neutrophils, macrophages, granulocytes, and epithelial cells	EC model (*in vitro*) PDAC tissue samples PDAC models (*in vitro*) GC models (*in vitro*) CRC stool samples CRC models (*in vitro* and *in vivo*)	Induction of Inflammation, cell proliferation, differentiation, motility, invasion, migration, cell apoptosis Antimicrobial activities Involvement in microenvironment formation	Upregulated in EC, PDAC, GC, CRC, HCC A prognostic marker for CRC and PDAC	(Refs. 21, 52–56)
S100A10	Calpactin–1	TLR4	Macrophages, granulocytes, and epithelial cells	PDAC tissue samples PDAC models (*in vitro*) GC models (*in vitro* and *in vivo*) CRC tissue samples CRC models (*in vitro*) HCC models (*in vitro* and *in vivo*)	Induction of cell proliferation, invasion, migration, cell apoptosis Involvement in microenvironment formation Interaction with ANXA2	Upregulated in PDAC, GC, CRC, HCC A biomarker for PDAC and CRC	(Refs. 57–60)
S100A11	Calgizzarin	RAGE	Ubiquitous expression in various cell types	PDAC models (*in vitro* and *in vivo*) GC tissue samples GC models (*in vitro*) CRC tissue samples CRC models (*in vitro*) HCC tissue samples HCC models (*in vitro*)	Induction of cell proliferation, differentiation, and cell apoptosis Inhibition of ANXI	Upregulated in PDAC, GC, CRC, HCC	(Refs. 61–64)
S100A12	Calgranulin-C	RAGE, GPCR, SR	Granulocytes and monocytes	GC tissue samples	Induction of inflammation Antibacterial activity	Downregulated in GC	(Ref. 65)
S100A13	-	RAGE	Ubiquitous expression in cell types	PDAC models (*in vitro* and *in vivo*)	Induction of cell motility, invasion, migration	Upregulated in PDAC	(Ref. 66)
S100A14	-	RAGE	Epithelial cells and cancer cells	PDAC tissue samples PDAC models (*in vitro* and *in vivo*) CRC tissue samples HCC serum samples EC model (*in vitro*) GC tissue samples GC models (*in vitro* and *in vivo*)	Induction of cell differentiation, cell cycle, cell growth, cell motility, invasion, migration	Upregulated in PDAC, CRC (early stage), HCC Downregulated in EC, GC	(Refs. 11, 67–70)
S100A16	S100F	-	Epithelial, endothelial, cancer cells, and fibroblasts	PDAC tissue samples PDAC models (*in vitro*) GC tissue samples GC models (*in vitro* and *in vivo*) CRC tissue samples CRC models (*in vitro* and *in vivo*)	Regulation of cell proliferation, differentiation, migration, invasion, and EMT	Upregulated in PDAC, GC Downregulated in CRC A potential prognostic biomarker for CRC	(Refs. 71–73)
S100B	-	RAGE, FGFR	Lymphocytes, dendritic, and cancer cells	CRC tissue samples	Induction of cell cycle, cell growth, cell motility	Upregulated in CRC A poor prognostic marker for CRC	(Ref. 74)
S100P	S100E	RAGE	Epithelial and cancer cells	PDAC models (*in vitro* and *in silico*) GC tissue samples GC models (*in vitro*) CRC models (*in vitro*) HCC tissue samples	Inhibition of cell growth, cell motility, invasion, migration	Upregulated in PDAC, GC, CRC, HCC A prognostic marker for CRC and HCC	(Refs. 75–78)

Abbreviations: ANX, annexin; CRC, colorectal cancer, EC, esophageal cancer; EGFR, epidermal growth factor receptor; EMT, epithelial-mesenchymal transition; FGF1, fibroblast growth factor 1; FGFR, fibroblast growth factor receptor; GC, gastric cancer; GI tract, gastrointestinal tract; GPCR, G protein-coupled receptors; HCC, hepatocellular carcinoma; PDAC, pancreatic adenocarcinoma; RAGE, advanced glycation end products; RARα, retinoic acid receptor alpha; SR, scavenger receptor; TLR4, toll-like receptor4.

**Figure 2.**
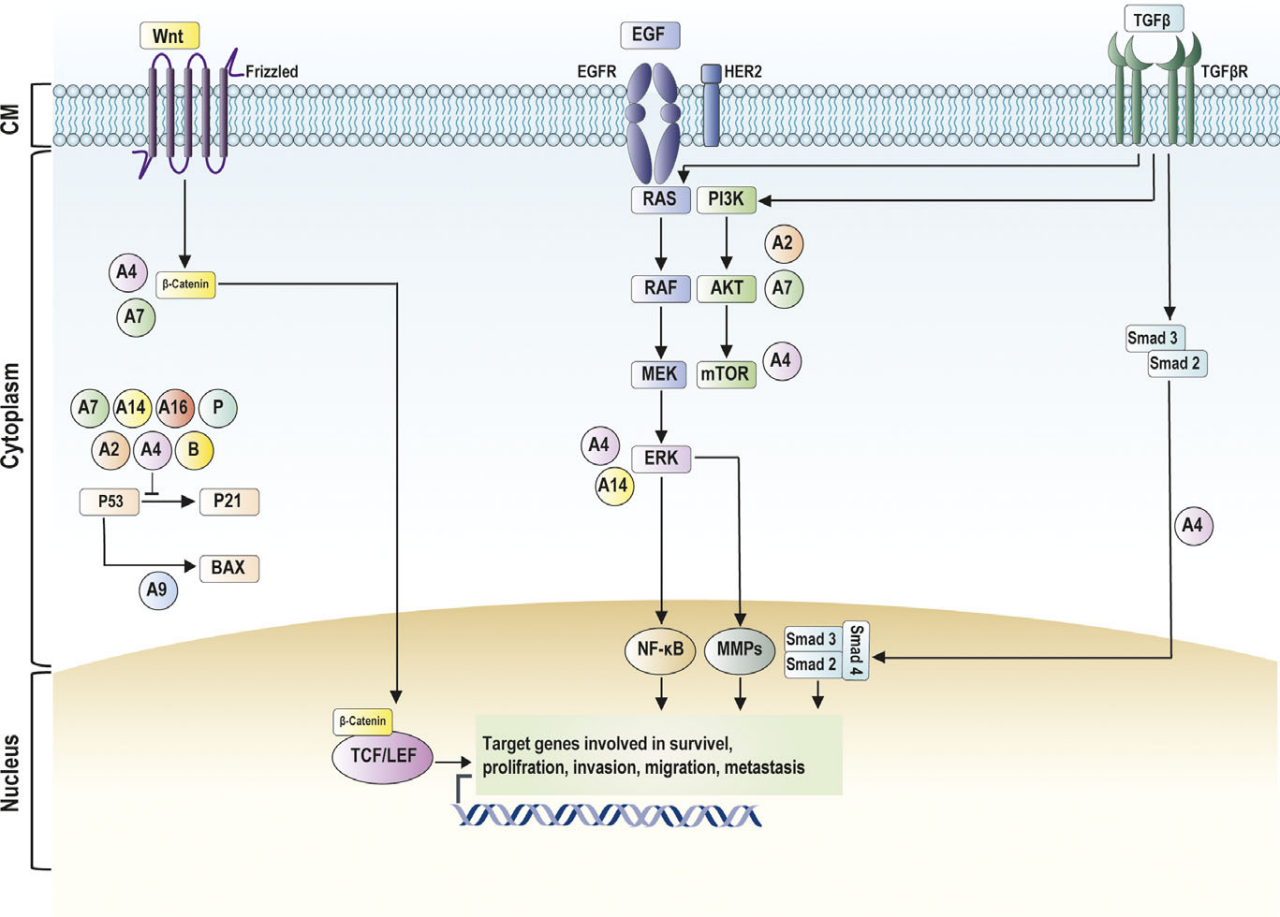
Schematic diagram of the intracellular activity of S100 proteins in signalling pathways involved in CRC. The Wnt signalling pathway is activated after binding Wnt to the Frizzled receptor, resulting in the translocation of β-catenin from the cytoplasm to the nucleus. In the nucleus, β- catenin interacts with TCF/LEF transcription factors. EGF binding to EGFR activates PI3K/AKT/mTOR and ERK signalling pathways. The TGF-β signalling pathway is activated after TGF-β binding to TGF-βR, leading to the formation of a trimeric complex of Smad. This complex is translocated to the nucleus, influencing gene expression related to cancer progression. The activation of these signalling pathways upregulates the expression of genes involved in cell survival, proliferation, differentiation, invasion, and metastasis. The binding of S100 proteins to P53 suppress p53 signalling pathway and pathways related to apoptosis. Abbreviations: AKT, protein kinase B; Bax, Bcl-2-associated protein x; EGFR, epidermal growth factor receptor; ERK, extracellular signalling-related kinase; HER2, human epidermal growth factor receptor 2; MEK, mitogen-activated protein kinase kinase; MMP, matrix metalloproteinase; mTOR, mammalian target of rapamycin; NF-κB, nuclear factor κB; PI3K, phosphatidyl inositol 3 phosphate; RAF, rapidly accelerated fibrosarcoma; RAS, rat sarcoma virus; TCF/LEF, T-cell factor/lymphoid enhancer factor; TGF-β: transforming growth factor-β; TGF-βR: transforming growth factor-β receptor; SMAD, suppressor of mothers against decapentaplegic; Wnt, wingless-related integration site.

S100 proteins play a role in the progression of CRC. The most important signalling pathways involved in cancer development are the wingless-related integration site (Wnt), phosphatidylinositol 3-kinase/protein kinase B/mammalian target of rapamycin (PI3K/AKT/mTOR), mitogen-activated protein kinase (MAPK), p53, TGF-β and EGFR signalling pathways (Ref. 105). These signalling pathways trigger the activation of various genes, such as β-catenin, p53, RAS and rapidly accelerated fibrosarcoma (RAF), which may be involved in CRC progression (Refs. 105, 106). Interestingly, some components of these signalling pathways regulate the expression of different S100 proteins. For example, previous studies have indicated that S100A4 is a direct transcriptional target of the Wnt/β-catenin/TCF-mediated signalling pathway. Interestingly, inhibition of the expression of β-catenin can lead to reduced levels of S100A4 in *in vitro* and *in vivo* CRC models (Refs. 107, 108). S100A6 is also transcriptionally regulated by β-catenin in CRC cells (Ref. 19), which stimulates tumour cell proliferation and migration (Ref. 15). EGF stimulates the expression of S100 proteins, such as S100A2, S100A7 and S100A9 (Refs. 109–111). P53 can interact with the promoters of S100 proteins, such as S100A2, S100A4, S100A9 and S100B, and increase their expression levels (Refs. 101, 112–115). In addition, intracellular S100 proteins can function in the regulation of key cellular factors and drivers of cancer, e.g., NF-κB, Wnt/β-catenin, AKT, p53 and MAPK cascades (including extracellular signal-regulated kinase 1/2 (ERK1/2), c-Jun N-terminal kinase (JNK), p38 kinases) (Figure 2). The activation of these signalling pathways by S100 proteins has been shown in different GI cancers (Table 2).

**Table 2.** Signalling pathways activated by S100 protein in GI tract cancers

Signalling pathways	S100 protein	Setting	Regulatory activity	Function	References
Wnt signalling pathway	S100A4	GC (Human)	Upregulation of β-catenin Downregulation of E-cadherin	Promotion of cell migration and metastasis	(Ref. 91)
	S100A6	PDAC (*in vitro*)	Upregulation of vimentin and β-catenin Downregulation of E-cadherin expression	EMT induction Promotion of cell proliferation, migration and invasion	(Ref. 92)
		GC (*in vitro*)	Regulation of β-catenin degradation and transcriptional activation of TCF/LEF	Inhibition of cell proliferation and tumourigenesis by affecting β-catenin degradation	(Ref. 93)
	S100A7	SCCOC (*in vitro*)	Downregulation of the β-catenin/TCF pathway	Inhibition of tumour growth and invasion Tumour suppressor activity	(Ref. 94)
	S100A8/S100A9	CRC (*in vitro*)	Regulation of expression of β-catenin and its target genes c-myc and MMP7	Promotion of viability and migration	(Ref. 95)
	S100A14	GC (*in vitro* and *in vivo*)	Upregulation of E-cadherin	Negative correlation with cell migration and invasion Inhibition of cell metastasis	(Ref. 67)
PI3K/AKT signalling pathway	S100A4	CRC (*in vitro*)	Activation of the PI3K/AKT/mTOR/p70S6K signalling pathway	Induction of cell migration Upregulation of VEGF Downregulation of E-cadherin	(Ref. 32)
	S100A10	GC (*in vitro* and *in vivo*)	Modulation of the Src/ANXA2/AKT/mTOR signalling pathway	Induction of cell proliferation Suppression of cell apoptosis	(Ref. 57)
		HCC (*in vitro* and *in vivo*)	Activation of ANXA2/AKT/mTOR pathway	Induction of cell proliferation	(Ref. 96)
	S100A11	PDAC (*in vitro*)	Activation of RAGE-MyD88-mTOR-p70 S6 kinase signalling Upregulation of PI3K/AKT signalling pathway	Upregulation of pro-survival pathways Promotion of cell proliferation	(Refs. 34, 97)
		HCC (*in vitro*)	Activation of AKT and ERK signalling pathways	Induction of cell proliferation, migration, invasion, and EMT	(Ref. 63)
	S100A16	PDAC (*in vitro* and *in vivo*)	Phosphorylation of AKT and ERK1/2	Promotion of cell proliferation and invasion in an FGF19-dependent manner	(Ref. 71)
MAPK signalling pathways	S100A6	CRC (*in vitro* and *in vivo*)	Activation of ERK and p38 MAPK pathways	Promotion of cell proliferation and migration, and tumour growth *in vivo*	(Ref. 15)
	S100A9	HCC (*in vitro*)	Phosphorylation of p38 and ERK1/2	Promotion of cell proliferation and invasion	(Ref. 98)
	S100A8/A9	Colon (*in vivo*)	Activation of MAPK and NF-κB signalling pathways	Promotion of leukocyte recruitment, angiogenesis, tumour migration, and wound healing Formation of premetastatic niches	(Ref. 99)
	100A8, S100A9, and S100A8/A9	PDAC (*in vitro*)	Activation of MAPK and NF-κB signalling at low concentrations	Promotion of tumour growth, cell survival, and metastasis	(Ref. 100)
EGFR signalling pathway	S100A10	HCC (*in vitro* and *in vivo*)	Regulation of AKT and ERK signalling via activating EGFR	Promotion of EMT	(Ref. 60)
		PDAC (*in vitro* and *in vivo*)	Activation of cellular invasiveness via KRAS signalling	Promotion of cell proliferation and invasion	(Ref. 58)
P53 signalling pathway	S100A6	HCC (*in vitro*)	Suppression of p53 protein expression by promoting the ubiquitination of p53 via the proteasome-dependent degradation pathway	Promotion of cell proliferation and migration	(Ref. 48)
	S100A9	EC (*in vitro*)	Interaction with p53	Regulation of p53 apoptosis pathway	(Ref. 101)
	S100A14	EC (*in vitro*)	Regulation of the expression and function of MMP2 in a p53-dependent manner	Promotion of cell motility and invasiveness	(Ref. 102)
TGF-β signalling pathway	S100A2	PDAC (*in vitro* and *in vivo*)	Activation of TGF-β/Smad2/3 signalling pathway Upregulation of SMAD4 expression	Promotion of cell proliferation, migration, and differentiation EMT induction	(Ref. 103)
	S100A4	GC (*in vitro* and *in vivo*)	Activation of (TGF)-β/Smad2/3 signalling pathway	Promotion of EMT process	(Ref. 104)

Abbreviations: AKT, protein kinase B; ANXA2, annexin A2; c-myc, cellular myelocytomatosis oncogene; CRC, colorectal cancer, EC, esophageal cancer; EGFR, epidermal growth factor receptor, EMT, epithelial mesenchymal transition; ERK1/2, extracellular signal-regulated kinase ½; FGF19, fibroblast growth factor 19; GC, gastric cancer; GI tract, gastrointestinal tract; HCC, hepatocellular carcinoma; KRAS, kirsten rat sarcoma viral oncogene; MAPK, mitogen-activated protein kinase; mTOR, mammalian target of rapamycin; MyD88, myeloid differentiation primary response protein 88; PDAC, pancreatic adenocarcinoma; PI3K, phosphatidylinositol 3-kinase; MMP, matrix metalloproteinase; NF-κB, nuclear factor kappa B; RAGE, advanced glycation end products; TCF/LEF, T-cell factor/lymphoid enhancer factor; TGF-β, transforming growth factor beta; TNF-α, tumour necrosis factor alpha; SCCOC, squamous cell carcinoma of the oral cavity; Wnt, wingless-related integration site.

## Role of S100 proteins in inflammation

Inflammation is a normal pathological process that is activated by infections at a specific tissue or organ site, but it can also be activated in disease states. Examples include microbial infections, such as *Helicobacter pylori* (Ref. 116), metabolic disorders, such as diabetes and obesity (Ref. 117), autoimmune disorders, such as inflammatory bowel disease (IBD) (Ref. 118), and environmental factors, such as heavy smoking (Ref. 119). Although cancer is multifactorial in origin, evidence has accumulated that chronic inflammatory conditions likely affect the development of various cancers, including gastric, liver, colorectal and pancreatic cancers (Refs. 21, 28, 54, 100). Chronic inflammation enhances the risk of tumourigenesis, metastasis, and treatment resistance in CRC (Refs. 28, 120, 121). For example, patients with IBD are at a higher risk of developing CRC because similar signalling pathways that mediate cell proliferation, survival, migration and angiogenesis are chronically activated (Ref. 122).

**Figure 3.**
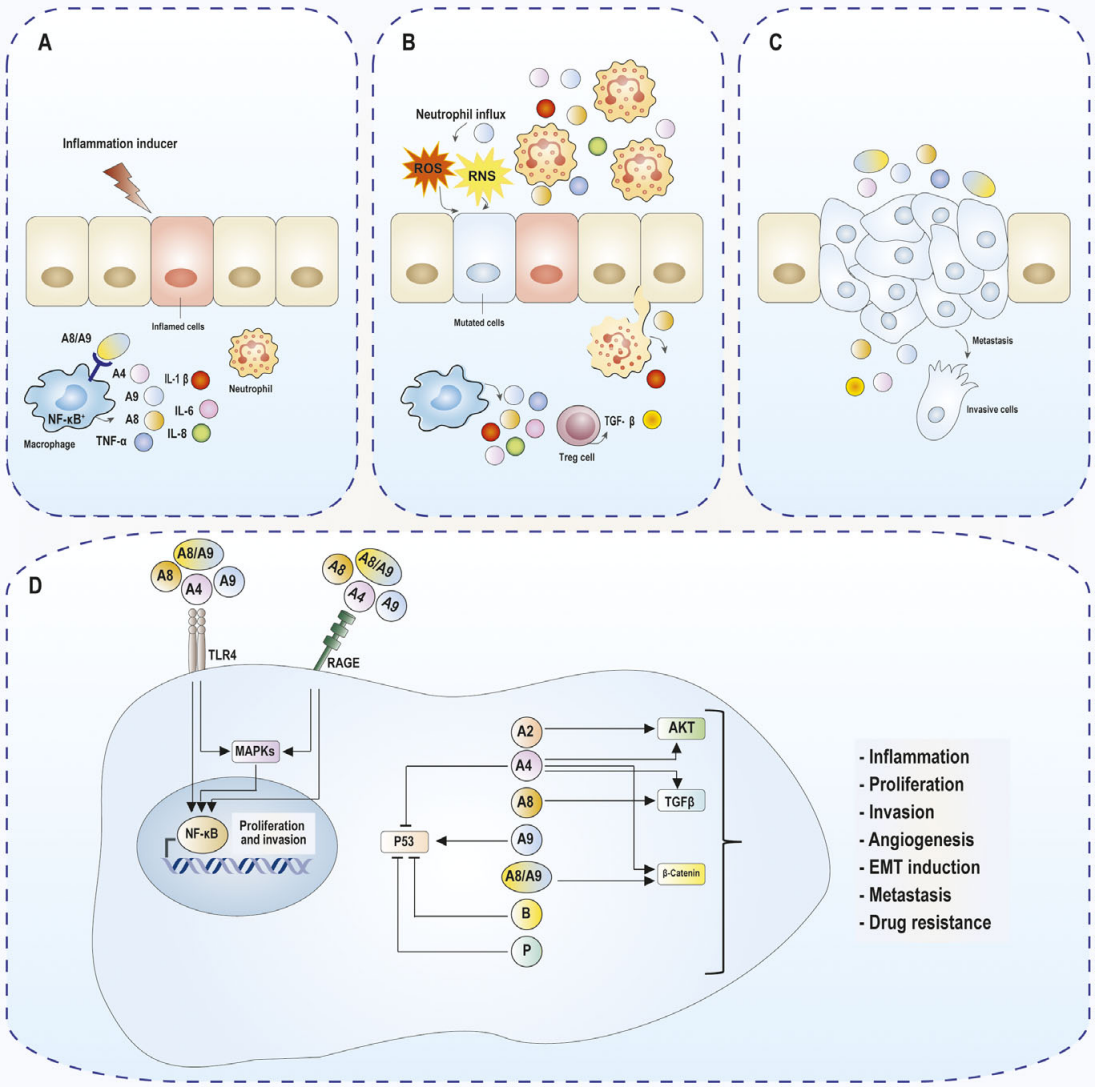
Schematic overview of the potential mechanisms of extracellular and intracellular S100 proteins during CRC progression. (A) inflammation inducers activate neutrophils and monocytes, leading to increase the release of S100 proteins such as S100A8 and S100A9. These proteins are the ligands of TLR4, which activates the NF-κB signalling pathway and elevates cytokine production, resulting in increases inflammation in epithelial cells. (B) Overexpression of S100A8 and S100A9 activates various immune cells (e.g., neutrophils, macrophages, T cells), leading to excessive production of cytokines. The increased expression of S100 proteins and cytokines such as TNF-α, IL-6, IL-1β, and IFN-γ stimulates the production of ROS and RNS, resulting in the induction of mutagenesis in premalignant cells. (C) S100 proteins and cytokines releases from cells can directly or indirectly affect cell proliferation, invasion, and metastasis. (D) Extracellular S100 proteins such as S100A4, S100A8, S100A9, and S100A8/S100A9 activate NF-κB and MAPK signalling and subsequently promote CRC cell survival and proliferation. Intracellular S100 proteins, such as S100A2, S100A4, S100A8, S100A9, S100A8/S100A9, S100B, and S100P, regulate signalling pathways related to cancer progression such as AKT, TGF-β, β-catenin, and P53, which are involved in the cell growth, proliferation, invasion or metastasis of CRC. Abbreviations: AKT, protein kinase B; IL, interleukin; MAPK, mitogen-activated protein kinase; NF-κB, nuclear factor κB; RAGE, advanced glycosylation end-product receptor; ROS, reactive oxygen species; RNS, reactive nitrogen species; TGF-β: Transforming Growth Factor-β; TNF-α, tumour necrosis factor alpha.

Among S100 proteins, S100A8 and S100A9 homodimers and heterodimers (S100A8/S100A9) are known as inflammation biomarkers (Refs. 24, 123). These proteins play decisive roles in the development of certain inflammatory processes (Refs. 20, 124). A role for S100A8/S100A9 in the context of an increased risk of CRC in patients with IBD has been proposed (Ref. 124). S100A8, S100A9 and S100A8/S100A9 are ligands for TLR4, which activate the NF-κB signalling pathway and elevate cytokine production (Refs. 20, 125). In addition to interacting with TLR4, S100A4, S100A7, S100A8/S100A9 and S100B bind to RAGE, activate NF-κB signalling and MAPK cascades, and increase the production of cytokines and chemokines (Figure 3A) (Refs. 21, 28, 51, 82). The activation of these molecules triggers immune responses via leukocyte recruitment, leading to the induction of immune cell-related factors and the promotion of a self-amplifying feedback cycle through the increase in the expression levels of different cytokines, including interleukin (IL)-1β, IL-6 and tumour necrosis factor alpha (TNF-α) (Refs. 20, 27, 125). These cascades alter pathways related to cell proliferation and growth factors that promote cancer progression (Ref. 105).

S100A4 and S100B can bind to p53, leading to decreased p53 binding of DNA and transcriptional activity, thereby compromising the tumour-suppressor activity of p53 (Refs. 114, 126). The loss of functional p53 induces the activation of NF-κB signalling and facilitates the secretion of Wnt ligands, which subsequently increase the expression of inflammatory markers and facilitate systemic neutrophilic inflammation (Ref. 127). These finding suggest that S100 proteins can stimulate cytokine secretion through different cancer-related signalling pathways and may trigger chronic inflammation, thereby facilitating tumour progression.

## Impact of S100 proteins on promoting tumourigenesis in CRC

S100 proteins exhibit a range of biological activities during cancer development and can act as mediators in cancer progression (Ref. 17). Moreover, the modes of action of S100 proteins can be altered according to the stage of cancer. The regulatory roles of S100 proteins in the progression of CRC are discussed in the following sections.

### Role of S100 protein in CRC initiation

Tumour initiation is the initial stage of tumourigenesis. At this stage, irreversible genetic changes caused by oncogenic factors transform normal cells into malignant cells (Ref. 128). S100A1, S100A8 and S100A9 stimulate the production of reactive oxygen species (ROS) and reactive nitrogen species (RNS) (Refs. 26, 29). The increase in these factors may lead to the activation of oxidative stress, resulting in damage to different biological macromolecules, including proteins, nucleic acids, and lipids (Ref. 129). The most sensitive target of oxidative stress is DNA, in which case ROS and RNS can cause DNA damage and mutations by incusing oxidation, halogenation, deamination, lipid peroxidation-derived adducts, and single- or double-strand breaks (Ref. 130). In addition, the binding of extracellular S100A1, S100A4, S100A6, S100A8/S100A9, S100A11, S100A12, S100A14, S100B, and S100P to TLRs and RAGE and activation of cellular signalling pathways resulting in the release of cytokines from inflammatory cells leads to ROS generation (Refs. 15, 29, 31, 82, 131) and may contribute to the induction of mutagenesis in premalignant cells (Figure 3B). In summary, S100 proteins are implicated in cancer initiation by stimulating oxidative stress and inducing the expression of inflammatory cytokines.

### Role of S100 proteins in CRC progression

The influence of pro-tumour factors and other specific conditions on damaged cells subsequently leads to the development of pre-malignant cells, which is known as the tumour promotion stage (Ref. 128). At this stage, cells undergo malignant conversion, resulting in aberrant proliferation to form the primary tumour and enter the stage of tumour progression (including tumour infiltration, invasion, and metastasis) (Figure 3C) (Refs. 105, 132). S100A2, S100A4, S100A6, S100A7, S100A8/S100A9 and S100B, either as intracellular or extracellular factors, may exhibit malignancy-promoting effects and may be associated with the regulation of cell cycle progression, proliferation, migration, and local invasion in cancers (Refs. 15, 33, 51, 95, 107, 133).

The effects of S100 proteins on CRC progression have been investigated, with a focus on S100A4, S100A8, S100A9 and S100A8/S100A9 (Figure 3D). S100A4 plays a role in different cancer stages, including cell motility, adhesion, proliferation, angiogenesis, invasion, cell survival and metastasis. It is associated with CRC progression by modulating the signalling pathways of NF-κB, MAPKs, PI3K/AKT/mTOR and EGFR (Refs. 28, 32). The interaction of S100A4 with p53 may contribute to tumour growth and metastatic progression (Ref. 114). S100A8 and S100A9, as homodimers or heterodimers, may affect tumour cell growth depending on their concentrations (Refs. 95, 101). Low concentrations of extracellular S100A8/S100A9 can promote cell growth by interacting with RAGE or TLR4 and subsequently activating MAPKs and the NF-κB signalling pathway, whereas high concentrations of S100A8/S100A9 can activate p53 transcriptional activity, presumably inducing p53-dependent cellular apoptosis (Ref. 101). Notably, overexpression of S100A8/S100A9 in certain tissues correlates with inflammation and appears to be an initial step in the transformation of neoplastic lesions during CRC progression (Ref. 79), suggesting a biological association between inflammation and neoplastic transformation in CRC.

Roles for other S100 proteins in CRC development have been demonstrated in several studies. For example, S100A2 activates the PI3K/AKT signalling pathway and upregulates glucose transporter 1 (GLUT1) expression in CRC, which induces glycolytic reprogramming and consequently increases tumour proliferation (Ref. 134). S100A3 appears to play a role in the occurrence and progression of various cancers; S100A3 overexpression may be linked to tumourigenesis and progression of CRC (Ref. 41). S100A6 is overexpressed in CRC tissue and may play a role in tumour promotion (Ref. 15). S100A14 is overexpressed in colon tissue (Ref. 135) and may play roles in different cellular processes, including cell growth, motility and adhesion in CRC (Ref. 11). Low concentrations of extracellular S100A14 are involved in cell proliferation by binding to RAGE; however, S100A14 expression is inversely correlated with CRC progression (Refs. 11, 136). Notably, S100A14 expression appears to be related to the expression of S100A1, S100A13 and S100A16, which are closely mapped to the 1q21 chromosomal region (Ref. 135). S100P inactivates p53 and promotes cell proliferation in CRC, contributing to cytotoxic therapy resistance (Ref. 137). S100P expression is also correlated with clinical staging, recurrence and lymph node metastasis (Ref. 75). However, the specific mechanisms underlying the expression of these proteins in CRC have not been fully elucidated.

### Role of S100 proteins in TME formation in CRC

S100 proteins impact not only tumour promotion but are also closely related to the formation of tumour microenvironment (TME) by activating various immune cells (e.g., macrophages, T cells, B cells, etc.), cytokines, and chemokines, further supporting the role of these proteins in tumour progression (Refs. 53, 138, 139). The TME is formed as sites where cancer cells are in a network of stromal cells, including inflammatory immune cells, vascular cells and fibroblasts altogether (Ref. 140). These proteins can affect TME formation by excessive production of pro-inflammatory cytokines and chemokines, recruitment of immune cells and angiogenesis induction (Refs. 28, 30, 140). Among the S100 family proteins, S100A4, S100A8 and S100A9 are closely related to the TME by upregulating cytokines and chemokines and inducing immune cell infiltration in tumours (Refs. 28, 99, 138, 139). These proteins can directly or indirectly activate the NF-κB signalling pathway, which is always present in the TME. In addition, these proteins may play crucial roles in the inactivation of tumour suppressors, such as TP53, which regulates cellular homeostasis and exhibits transcriptional antagonism with NF-κB. This process increases the expression of NF-κB-dependent inflammatory genes (Ref. 141).

S100 proteins may also play a decisive role in immune cell infiltration in TME. For example, Hatthakarnkul et al. (Ref. 142) found that S100A2 expression was correlated with increased infiltration of CD163 M2-like macrophages in the tumour, suggesting that S100A2 contributes to immune activation (Ref. 142). In another study, Kazakova et al. (Ref. 143) analysed human CRC tissues and revealed that S100A4 was expressed by stromal compartments, particularly tumour-associated macrophages (TAMs) and showed a strong correlation with macrophage infiltration in CRC tumours (Ref. 143). In addition, TAMs can be characterised by the upregulation of chemokine genes, such as IL-1β, IL-6 and IL-8, and S100 genes such as S100A8/S100A9 and S100A9 in primary CRC, resulting in the recruitment and regulation of immune cells (Ref. 144). Notably, the increased levels of IL-6 and IL-8 in the TME can upregulate the expression of S100A8/S100A9 in tumour-infiltrated myeloid cells, the differentiation of myeloid cells into S100A8/S100A9-expressing myeloid-derived suppressor cells (MDSCs) or M2 macrophages in the TME of CRC (Ref. 145). Further studies are needed to determine the role of S100 proteins in the CRC microenvironment.

### Role of S100 proteins in CRC metastasis

Metastasis is a complicated process, and different factors exert effects on the progression of metastasis (Ref. 6). Recent studies have supported the involvement of different S100 proteins in CRC metastasis (Refs. 45, 52, 71, 146). The involvement of S100A4, S100A8 and S100A9 has been investigated in tumourigenesis, epithelial-mesenchymal transition (EMT), invasion and cell migration of CRC (Refs. 28, 52, 147). It is well documented that S100A4 overexpression is associated with increased metastatic potential in patients with CRC (Ref. 28), as S100A4 knockdown limits metastasis formation in CRC xenograft mouse models (Ref. 148). This protein can induce production of cytokines TNF-α and IL-1β, which regulate the expression of transcription factors, such as Twist and Slug, that induce EMT (Ref. 149). Moreover, S100A4 contributes to the recruitment of TGF-β-driven and -producing fibroblasts, leading to immune escape and tumour invasion in cancer (Ref. 104). Extracellular S100A8 and/or S100A9 are also involved in the formation of the pre-metastatic niche by upregulating chemokine expression, such as CXC motif chemokine ligand 1 (CXCL1) and C-C motif ligand 5 (CCL5) (Refs. 30, 150). At low concentrations, they may induce CRC cell invasion. In addition, these proteins upregulate the expression of different cytokines and may be involved in EMT formation (Refs. 30, 95). S100A8 promotes TGF-β-induced EMT and metastasis in CRC mouse models (Ref. 52), suggesting that S100A8 and upstream transcription factor 2 (USF2) expression is upregulated during TGF-β-induced EMT and cell migration and invasion in CRC cells.

Among other S100 proteins, S100A2 is involved in metastasis through the formation of the S100A2/ Karyopherin alpha 2 (KPNA2) cotransport complex, which promotes cell metastasis by modulating the tumour-associated nuclear transcription factor y subunit alpha (NFYA) (Ref. 151). S100A6 may contribute to invasion and metastasis in CRC cells by activating MAPK pathways (Ref. 15). S100A11 can interact with LIM and SH3 protein 1 (LASP1) and enhance its expression in CRC. The LASP1-S100A11 axis can activate of TGF-β1/SMAD pathway, which mediates EMT and CRC progression (Ref. 62). S100B is overexpressed in metastatic CRC and stimulates cell growth, invasion, and migration through interaction with thioredoxin-1 (Trx-1) and β-tubulin (Ref. 152). S100P is a transcriptional target gene for the transcriptional regulator of metastasis associated in colon cancer 1 (MACC1), which is a well-known driver of metastasis formation in CRC (Refs. 146, 153). Schmid et al. (Ref. 153) reported that S100P boosts the metastatic potential of CRC cells *in vitro* and *in vivo* via MACC1 (Ref. 153). Although different studies have confirmed the role of S100 proteins in the development of metastasis in CRC, the specific functions of most S100 proteins have not yet been fully characterised.

### Roles of S100 proteins in drug resistance

Drug resistance is a complex process in which malignant cells become insensitive to anticancer drugs (Ref. 154). S100 proteins are differentially expressed in drug-resistant tumours (Ref. 155) and may be involved in drug resistance in cancers. Intracellular S100 proteins intensify drug resistance by regulating different signalling pathways involved in cancer, such as reducing Ca^2+^ levels, inactivating p53, promoting annexin A2 (ANXA2) phosphorylation, regulating apoptosis and autophagy, elevating the expression of drug transporters (MDR-1, MRP-1 and LRP), activating cancer stem cells (CSCs) and interacting with non-coding RNAs (Refs. 17, 155). In addition, the binding of extracellular S100 proteins to RAGE or TLR4 can activate NF-κB signalling pathways and promote EMT, and the activation of these processes plays a critical role in the induction of chemo-drug resistance (Refs. 155–157).

Roles for different S100 proteins in inducing resistance in CRC cells to various chemotherapeutic drugs have been proposed. Chemotherapeutics may cause differential expression of S100 proteins; the expression levels of S100A2, S100A3, S100A4 and S100A10, notably upregulated in CRC tissues compared with healthy controls, are over 2-fold lower in 5-FU-resistant colon cancer cells (Ref. 158). S100A4 expression is upregulated in different drug-resistant colon cancer cells, including those resistant to methotrexate (Ref. 85) and cisplatin (DDP) (Ref. 159). Boye et al. (Ref. 160) reported that 5-FU sensitivity is not significantly correlated with S100A4 levels, since no difference in 5-FU sensitivity was observed among different groups, including overexpressed S100A4, S100A4-knockdown, and control CRC cells (Ref. 160). A strong correlation between S100A10 expression and oxaliplatin resistance was reported. Suzuki et al. (Ref. 161) found that S100A10 in combination with ANXA2 can mediate oxaliplatin resistance in *in vitro* CRC models (Ref. 161). Notably, S100A10 expression levels were not correlated with 5-FU-sensitive CRC cells, demonstrating that deregulated S100A10 expression is more specific to oxaliplatin than to 5-FU. Additionally, Fenouille et al. (Ref. 162) found that the expression levels of S100A10, ANXA2, and calpain 2 may be involved in irinotecan resistance, since their expression levels are significantly higher in irinotecan-resistant HT-29 CRC cells compared to irinotecan-sensitive cells (Ref. 162). S100P was upregulated in an oxaliplatin-resistant cell line (Ref. 163), whereas it was downregulated in a doxorubicin-resistant cell line (Ref. 84). Previous studies have demonstrated that S100P interferes with the interaction between p53 and its corresponding repressor HDM2, disrupting the normal regulatory mechanisms of p53-HDM2 interaction, which may facilitate chemotherapy resistance. Further studies are needed to explore the precise roles of S100 proteins in processes involved in drug resistance.

## Association of S100 proteins and molecular subtypes of CRC

Few studies have investigated the relationship between S100 proteins and different CRC subtypes. The multi-omics analyses revealed that the S100 protein expression levels could be correlated with distinct gene expression patterns in CRC. For example, Kundu et al. (Ref. 164) demonstrated that the expression levels of S100A2 and S100A10 were higher in KRAS-mutated CRC cells compared to BRAF-mutated cells (Ref. 164). Previous evidence has demonstrated that CMS2 and CMS3 tumours harbor high mutations in KRAS (Ref. 165). Hence, S100A2 and S100A10 expression may be positively correlated with these CRC subtypes. S100A4 plays a critical role in the deregulation of Wnt/β-catenin pathways in CRC. Lee et al. (Ref. 166) found that although β-catenin nuclear expression was increased in MSS tumours compared with MSI-H tumours, there was no significant difference between expression levels of S100A4 in MSI-H and MSS CRC papulation (Ref. 166). Another study indicated that the prognostic significance of S100A4 mRNA expression does not depend on cancer type (Ref. 143). A previous study demonstrated that the gene signature of M11 (S100B + MMP12 + CD68+ TAMs) could be associated with good prognosis in MSS (CMS2/CMS3) tumours, whereas it shows little correlation with outcomes in MSI (CMS1) CRC tumours (Ref. 144), demonstrating S100B may be associated with CMS2 and CMS3 subtypes. Importantly, the profile of IBD-related CRC belongs to the CMS4 tumours (Ref. 167). Accordingly, S100A8 and S100A9 are known as biomarkers for IBD and may be associated with CMS4. In addition, a single-cell RNA sequencing analysis demonstrated that S100A9+ macrophage contributes to immunosuppressive TME and ICI resistance in patients with C3 subtype MSI-H CRC, suggesting that S100A9 may be associated with CMS1 (Ref. 138). Future studies should investigate the functions of S100 proteins in different CRC subtypes.

## S100 proteins as biomarkers for CRC

S100 proteins can be considered as potential biomarkers for different types of cancer (Refs. 47, 54, 70, 74). Mounting evidence demonstrates that the expression of S100 proteins is altered in different stages of CRC and holds promise as a stage-specific biomarker. Zeng et al. (Ref. 168) reported that the mRNA levels of S100A2, S100A3, S100A9, S100A11 and S100P were upregulated and S100B was downregulated in CRC tissues compared with normal colon mucosa (Ref. 168). Hatthakarnkul et al. (Ref. 142) demonstrated that low cytoplasmic S100A2 could exhibit a prognostic role in CRC and the relationship between S100A2 and immune infiltration in CRC (Ref. 142). The expression level of S100A2 may be a potential biomarker in several types of cancer, such as pancreatic cancer and endometrial carcinoma (Refs. 13, 37). However, further research is warranted to clarify the exact function of S100A2 in the development of CRC. S100A4 is not only introduced as a biomarker for detecting metastatic states in patients with a high risk of metastatic development, but it is also involved in mediating metastatic processes; therefore, it may be a target for the diagnosis and monitoring of CRC. Destek and Gul. (Ref. 45) noted that the S100A4 expression in colon cancer tissue samples can be linked to tumour localisation, malignant tumour (TNM) staging, increased aggressiveness and poor survival, suggesting that S100A4 may be a prognostic marker for TNM staging and poor survival (Ref. 45). S100A8/S100A9 may also be a biomarker for detecting CRC. Different studies have demonstrated that the S100A8 and S100A9 overexpression can be linked to lymph node metastasis in CRC and may be considered as biomarkers for CRC metastasis (Ref. 169). Notably, Lehmann et al. (Ref. 170) indicated that the majority of patients with CRC with T3 and T4 tumours had higher fecal S100A8/S100A9 levels than those with T1 and T2 tumours (Ref. 170). Overexpression of S100A10 may be a predictor for CRC recurrence based on its correlation with advanced-stage CRC (Ref. 171). S100A10 overexpression is significantly correlated with oxaliplatin resistance, and this protein exhibits a specific pattern in drug resistance (Ref. 161). Increased mRNA levels of S100A11 in the tissue from patients with advanced adenoma have been observed when compared with the healthy mucosa in those with advanced adenomatous polyps (Refs. 79, 172). S100A11 overexpression was related to poor disease-free survival (DFS) in CRC, suggesting that S100A11 expression may be involved in tumour metastasis and may be considered as a potential prognostic marker. S100A16 expression is negatively correlated with CRC prognosis because S100A16 could suppress proliferation, migration and invasion in different CRC cell models (Ref. 72). Thus, S100A16 may also serve as a prognostic biomarker of CRC (Refs. 72, 173). S100B is overexpressed in patients with liver metastases of CRC and can be used as a marker for predicting early relapse in patients with stage II and III postoperative colon cancer (Refs. 74, 83). S100P is overexpressed in patients with CRC and can be detected in serum and fecal samples. Higher levels of S100P in primary colorectal tumours and metastatic lesions compared with normal tissue have been previously reported in CRC (Refs. 153, 174). These studies suggest that S100P may be considered as a potential prognostic biomarker of metastasis-free survival in patients with CRC and a potential prognostic biomarker for primary tumours. However, other studies have not supported this finding, as there was no significant difference in the mRNA levels of S100P between different groups, including advanced adenoma patients, non-advanced adenoma patients, and controls (Ref. 79). Further studies are needed to select S100 proteins as potential biomarkers for CRC.

## S100 proteins as therapeutic targets for CRC

Currently, new targeted therapies have been introduced for cancer therapy, including natural and synthetic-molecule inhibitors, neutralising antibodies, peptide inhibitors, and microRNA (miRNA) mimics, some of which have already been approved or are undergoing clinical trials (Refs. 195, 196). Given the potential role of S100 proteins in the development of CRC, if these can be validated, they would serve as therapeutic targets to prevent CRC progression. To date, several studies have focused on the blockade of different S100 proteins in cancer states, most of which are limited to *in vitro* studies, and some data are controversial (Ref. 197). Most inhibitors introduced for S100 proteins in cancer therapy are presented in Table 3.

**Table 3.** Overview of potential S100 proteins-targeting inhibitors for treating GI tract cancers

Target	Inhibitor type	Inhibitor name	Current status	Cancer treatment	Mechanism of action	References
S100A2	Natural molecule	Delanzomib	Pre-clinical	CRC, HCC	Inhibition of S100A2-KPNA2 interaction Inhibition of tumour growth Preventing CRC metastasis	(Refs. 151, 175)
S100A4	Synthetic molecule	Niclosamide	Phase II	CRC, EC, PDAC, HCC	Inhibition of β-catenin/TCF complex Inhibition of cell proliferation Enhancement of chemotherapy efficacy Promotion of cell apoptosis via mTOR-dependent autophagy	(Refs. 176–179)
		Calcimycin	Pre-clinical	CRC	Inhibition of Wnt/β-catenin signalling phathway	(Ref. 107)
		Sulindac	Pre-clinical	CRC, EC, PDAC, HCC, GC	Inhibition of β-catenin expression Inhibition of inflammation Induction of cell apoptosis Inhibition of KRAS protein prenylation	(Refs. 108, 180–183)
		TFP	Pre-clinical	CRC, HCC	Inhibition of cell proliferation Induction of cell apoptosis Disabling interaction between S100A4 and myosinIIA	(Refs. 184, 185)
	Antibodies	Anti-S100A4 antibody	Pre-clinical	CRC	Inhibition of inflammation Inhibition of tumour growth	(Ref. 28)
	miRNAs	miR–187–3p	Pre-clinical	HCC	Inhibition of EMT induction Reduction in metastasis	(Ref. 186)
		miR–325–3p	Pre-clinical	CRC	Attenuation of bone resorption in bone metastasis of CRC	(Ref. 187)
		miR–296	Pre-clinical	CRC	Inhibition of EMT induction, migration, and invasion	(Ref. 188)
S100A8/A9	Antibodies	Anti-S100A9 antibody	Pre-clinical	CRC	Inhibition of Wnt and PI3K/AKT signalling pathways	(Ref. 67)
		Divalent peptide3A5	Pre-clinical	CRC	Inhibition of tumour progression and metastasis Inhibition of VEGF production	(Ref. 189)
		S100A8/A9-targeting peptide	Pre-clinical	CRC	Inhibition of EMT induction Inhibition of tumour growth	(Ref. 190)
S100B	Synthetic molecule	Pentamidine	Phase II (for PDAC) Pre-clinical (for CRC)	CRC, PDAC	Inhibition of interaction of S100B with p53 and restoring P53 activity	(Refs. 133, 191)
S100P	Synthetic molecule	Cromolyn	Pre-clinical	CRC, PDAC	Inhibition of interaction of S100P with RAGE	(Refs. 192–194)

*Abbreviations*: AKT, protein kinase B; CRC, colorectal cancer, EC, esophageal cancer; EMT, epithelial mesenchymal transition; TFP, trifluoperazine; GC, gastric cancer; GI tract, gastrointestinal tract; HCC, hepatocellular carcinoma; KPNA2, karyopherin alpha 2,KRAS, kirsten rat sarcoma viral oncogene, mTOR, mammalian target of rapamycin, PDAC, pancreatic adenocarcinoma; PI3K, phosphatidylinositol 3-kinase; RAGE, advanced glycation end products; TCF, T-cell factor; VEGF, vascular endothelial growth factor; Wnt, wingless-related integration site.

### Natural and synthetic molecules

Natural molecule inhibitors are classified as low-molecular-weight organic compounds that target important cancer-promoting proteins (Ref. 198). The inhibition of S100 proteins by small molecules has been previously reported. For CRC, it is documented that the interaction between S100A2 and KPNA2 mediates the transportation of the tumour-associated transcription factor NFYA, inhibits E-cadherin transcriptional activity, and subsequently facilitates metastasis. Han et al. reported that the use of a natural molecule, i.e., delanzomib, inhibits the S100A2-KPNA2 interaction and prevents CRC metastasis (Ref. 151). Accordingly, delanzomib is a promising treatment option for preventing the progression of CRC.

In recent years, different synthetic molecules with inhibitory activity against S100A4 have been introduced to suppress cancer progression. For example, niclosamide, an FDA-approved anti-parasitic drug, has shown promising results for the treatment of various cancers and is being examined in clinical trials for different solid tumours (Refs. 176–179). Niclosamide modulates β-catenin signalling and induces β-catenin degradation by inhibiting S100A4 transcription (Ref. 108). The safety and efficacy of niclosamide for patients with metastatic CRC are currently being evaluated in a phase II clinical trial (Ref. 199). Kortum et al. (Ref. 179) showed that the inhibition of S100A4 by niclosamide reduced cell proliferation, motility and migration *in vitro*, supporting the therapeutic value of targeting S100A4 as an anti-metastatic approach for CRC (Ref. 179). Another S100A4 inhibitor, sulindac, an FDA-approved nonsteroidal anti-inflammatory drug (NSAID), significantly downregulated the mRNA and protein levels of β-catenin and S100A4 in tumours and reduced tumour growth and metastasis formation in an animal model compared with controls. Additionally, sulindac treatment reduced β-catenin and S100A4 levels in liver metastases (Ref. 108). Calcimycin is an antibiotic drug that can inhibit the activity of S100A4. Sack et al. (Ref. 107) found that calcimycin dysregulated the Wnt/β-catenin pathway and restricted cell motility and metastasis induced by S100A4 in colon cancer cells (Ref. 107). These findings support S100A4 targeting as an effective approach for inhibiting the Wnt signalling pathway and demonstrate anti-tumour and anti-metastatic effects (Ref. 153). Trifluoperazine (TFP) is an FDA-approved antipsychotic drug and acts as an S100 protein inhibitor that disrupts the S100A4/myosin-IIA interaction. Qian et al. (Ref. 184) demonstrated that the administration of TFP reduced tumour growth and proliferation and inhibited the EMT phenotype in a CRC mouse model, suggesting the potential antitumour activity of TFP in CRC (Ref. 184).

Paquinimod and tasquinimod have been introduced as S100A8/S100A9 inhibitors that suppress RAGE and TLR4 signalling, respectively (Refs. 200, 201). A single-cell RNA sequencing analysis revealed the involvement of S100A9+ macrophages in patients with MSI-H (C3 subtype) that show resistance to anti-programmed death 1 (PD-1) antibody (Ref. 138). Interestingly, administration of PD-1 blockade and tasquinimod reversed dysfunctional immunotherapy response and enhanced immunotherapy efficacy by depleting S100A9+ macrophages *in vivo* (Ref. 138). These results support the potential value of targeting S100A9 as a therapeutic strategy for treating patients with PD-1-resistant MSI-H CRC.

Inhibition of S100B by pentamidine (PTM) disrupts the S100B/p53 interaction (Ref. 202), which promotes the expression of wtp53 and recovers p53-dependent cellular apoptosis in an *in vitro* colon cancer model (Ref. 133). Inhibition of S100P by cromolyn suppressed cell proliferation in CRC cell lines and attenuated tumour morphometric outcomes in an *in vivo* model of CRC by reducing tumour volume and weight compared with the control group. This suggests that the blockade of S00P by cromolyn may be a relevant anticancer approach for future therapeutic development (Ref. 192). However, this inhibitor binds other S100 proteins, such as S100A1, S10012 and S100A13 due to the high structural similarity of these proteins, and the consequences of the lack of S100 protein specificity have not been investigated.

### Neutralising antibodies

Neutralising anti-S100 antibodies have been introduced as novel therapeutic strategies for modulating different cellular processes, with S100A4, S100A8, S100A9 and S100P targeting by specific antibodies having been studied (Refs. 20, 28, 147, 203). However, few studies have investigated the application of anti-S100 antibodies against CRC. For instance, Zhang et al. (Ref. 147) used an anti-S100A9 antibody to suppress colitis-associated colon cancer in mice. This study indicated that treatment of CRC mouse models with anti-S100A9 antibody may suppress key pathways involved in CRC development, such as the Wnt and PI3K/AKT signalling pathways. It has been suggested that an anti-S100A9 antibody could decrease the risk of CRC development (Ref. 147). Additionally, Zhang et al. (Ref. 28) reported that an anti-S100A4 neutralising antibody reduced tumour incidence in a mouse model, suggesting that it may serve as an approach for the prevention and treatment of colon cancer (Ref. 28).

### Peptide inhibitors

In addition to antibodies, specific S100-blocking peptides have been introduced in an attempt to develop cancer therapies. For example, Deguchi et al. (Ref. 189) generated a multivalent peptide to inhibit the activity of S100A8 (divalent peptide3A5). Treatment of human colorectal tumour SW480 cells with this peptide diminished tumour progression and metastasis by preventing the activation of the TLR4-dependent pathway and suppressing VEGF production. Interestingly, administration of this peptide in a CRC mouse model improved the efficacy of bevacizumab (an anti-VEGF antibody) and attenuated lung metastasis (Ref. 189). Therefore, this peptide may be a treatment option for aggressive cancer. In another study, the application of an S100A8/S100A9-targeting specific peptide suppressed tumour growth and downregulated the expression of EMT-associated markers in CRC mouse models (Ref. 190).

### MicroRNA (miRNA) mimics

miRNAs belong to a class of small RNA molecules (approximately 18–24 nucleotides) that play critical roles in the regulation of different signalling pathways related to cancers, and miRNA mimics have been introduced as a pharmaceutical strategy for treating cancers (Ref. 204). Furthermore, miRNAs are involved in the expression of different S100 proteins (Refs. 187, 205, 206). Several studies have suggested that miRNA mimics may serve as effective agents for blocking S100 expression, most of which focus on targeting S100A4 as an effective strategy for preventing CRC. Two miRNAs, i.e., miR-187-3p and miR-149-3p, have been shown to decrease S100A4 expression and inhibit tumour cell proliferation, invasion and migration by blocking S100A4 (Refs. 206, 207). In addition, the miR-149 − 3p–S100A4–p53 axis contributes to induced drug resistance, since lower expression of miR-149 − 3p was observed in DDP-resistant colon cancer cells than in DDP-sensitive cells (Ref. 159).

In CRC cells, miR-325-3p/S100A4, miR-520c-3p/S100A4 and miR-296/S100A4 play important roles in carcinogenesis. Chengling et al. (Ref. 187) indicated that miR-325-3p can target S100A4, and overexpression of miR-325-3p efficiently attenuated bone resorption in bone metastasis of CRC (Ref. 187). He et al. (Ref. 188) found that miR-296 inhibited EMT, migration and invasion of CRC cells by mediating S100A4, demonstrating that miR-296 can be a potential target against CRC metastasis (Ref. 188). There are no data available on the application of miR-520c-3p to suppress S100A4 expression, but Mudduluru et al. (Ref. 205) demonstrated that epigenetic silencing of miR-520c induced the expression of S100A4 and subsequently promoted CRC progression, suggesting that miR-520c regulated S100A4 expression (Ref. 205). Although miRNAs have shown high effectiveness in targeting S100 proteins, systems with high efficacy for the miRNA delivery needs to be explored to reach an effective therapy in a clinical context. Further studies are needed to explore an effective approach for targeting S100 proteins with high efficacy for therapeutic interventions in CRC.

## Discussion

S100 proteins play critical roles in the development of CRC and can be considered as potential biomarkers and therapeutic targets for CRC management. Different S100 proteins, including S100A4, S100A8/S100A9, S100A10 and S100B, have shown promising results as biomarkers for CRC monitoring or assessing specific drug resistance (Refs. 74, 161, 169). Notably, most tests introduced for detecting S100 proteins are based on ELISA methods, and numerous commercial kits for measuring S100 proteins, such as S100A8/S100A9 and S100B, in biological fluids are already available, providing a non-invasive method for disease monitoring. Nonetheless, few studies have investigated the amount of S100 proteins in serum and fecal samples of patients with CRC, which are mostly limited by insufficient sample size. In addition, the precise expression level of these proteins in different CRC populations is not fully understood; thus, more comprehensive studies are needed to elucidate the exact relationship between S100 proteins in different CRC subtypes.

S100 proteins also show potential as therapeutic targets for CRC, with S100A4 showing the most promising results in CRC treatment (Refs. 139, 188, 199). Among the different S100A4 inhibitors, niclosamide has shown the most promise for suppressing the development of different cancers (Ref. 199). Further research is required to clarify the efficacy of this inhibitor for treating CRC. It should be noted that the efficacy of targeting S100 proteins as a therapeutic approach may be limited due to cross-reactivity between different S100 proteins, their long half-life and insufficient steady-state protein levels (Ref. 197). In the future, further efforts should focus on improving the specificity and pharmacological effects of S100 inhibitors (e.g., biological half-life and affinity). In addition, evidence of the precise role of other S100 proteins in CRC progression is limited; therefore, a better understanding of the roles of extracellular and intracellular S100 proteins can help identify new therapeutic targets. The application of new technologies and bioinformatics tools can help reveal the functional roles of S100 proteins in cellular processes and signalling cascades in multiple cell types and provide a better understanding of the importance of S100 proteins in CRC clinical diagnosis, prevention and treatment.

The investigation of the precise roles of S100 in different CMSs of CRC may provide new insights into the predation and treatment of patients with CRC, as CMSs have shown different molecular features, prognosis, and drug responses (Ref. 208). In this regard, investigating the importance of specific S100 proteins based on cancer grades and subtypes may provide insights into better stratifying patients with distinct TME properties and exploring personalised therapeutics. For example, S100 proteins, such as S100A8/S100A9, are known as biomarkers for inflammatory diseases and may be associated with CMS4 tumours (Ref. 167). This CRC subtype is associated with the poorest prognosis and worse overall and relapse-free survival rate than the other three subtypes; thus, S100A8/S100A9 may be a potential diagnostic and therapeutic target for CRC patients with CMS4 tumours. Therefore, future studies should be conducted to investigate both the genetic profile and immunomodulatory functions of S100 proteins in patients with CRC.

In conclusion, S100 proteins, especially S100A4, S100A8, S100A9, S100B, S100A8/S100A9 and S100A10, can play critical roles in CRC initiation, progression and drug resistance by activating cancer-signalling pathways. Dysregulated expression of these proteins in CRC tissues can be considered as a potential biomarker for disease monitoring and specific drug resistance and developing therapeutic agents for CRC management. However, most recent studies on the importance of S100 proteins in CRC progression have focused on basic and pre-clinical research. Further efforts are needed to explore the precise role of these proteins, particularly in different CMSs of CRC and assess their potential for developing new diagnostics and personalised therapeutics targeting specific CRC and drug-resistant tumours.

## Abbreviations

AKT: protein kinase B
ANXA2: annexin A2
ANXA6: annexin A6
AP-1: activator protein 1
Bax: Bcl-2-associated protein x
CCL5: C-C motif ligand 5
CIN: chromosomal instability
CMS: consensus molecular subtypes
c-myc: cellular myelocytomatosis oncogene
CRC: colorectal cancer
CRCSC: colorectal cancer subtyping consortium
CSCs: cancer stem cells
CXCL1: CXC motif chemokine ligand 1
DDP: cisplatin
DFS: disease-free survival
EC: esophageal cancer
EGFR: epidermal growth factor receptor
ELISA: enzyme-linked immunosorbent assay
EMT: epithelial mesenchymal transition
ERK: extracellular signalling-related kinase
FGF: fibroblast growth factor
FGFR: fibroblast growth factor receptor
GC: gastric cancer
GI tract: gastrointestinal tract
GLUT1: glucose transporter 1
GPCR: G protein coupled receptor
HCC: hepatocellular carcinoma
HER2: human epidermal growth factor receptor 2
IBD: inflammatory bowel disease
IL: interleukin
JNK: c-Jun N-terminal kinase
KPNA2: karyopherin alpha 2
KRAS: kirsten rat sarcoma viral oncogene homologue
LASP1: LIM and SH3 protein 1
MACC1: metastasis associated in colon cancer 1
MAPK: mitogen-activated protein kinase
MDR: multiple drug resistance
MEK: mitogen-activated protein kinase kinase
miRNA: microRNA
MMP: matrix metalloproteinase
MMR: mismatch repair
MSI-H: microsatellite instability-high
MSS: microsatellite stability biomarker
mTOR: mammalian target of rapamycin
MyD88: myeloid differentiation primary response protein 88
NFYA: nuclear transcription factor y subunit alpha
NF-κB: nuclear factor κB
PD 1: programmed death 1
PDAC: pancreatic adenocarcinoma
PDSs: pathway-derived subtypes
PI3K: phosphatidyl inositol 3 phosphate
PTM: pentamidine
RAF: rapidly accelerated fibrosarcoma
RAGE: receptor for advanced glycation end products
RARα: retinoic acid receptor alpha
RNS: reactive nitrogen species
ROS: reactive oxygen species
SCCOC: squamous cell carcinoma of the oral cavity
SCNAs: somatic copy number alterations
SMAD: suppressor of mothers against decapentaplegic
SR: scavenger receptor
TAMs: tumour-associated macrophages
TCF/LEF: T-cell factor/lymphoid enhancer factor
TFP: trifluoperazine
TGF-β: transforming growth factor-β
TGF-βR: transforming growth factor-β receptor
TLR4: toll-like receptor 4
TME: tumour microenvironment
TNF-α: tumour necrosis factor alpha
TNM: malignant tumours
Trx-1: thioredoxin-1
USF2: upstream transcription factor 2
VEGF: vascular endothelial growth factor
Wnt: wingless-related integration site
